# Effect of Supination Versus Pronation in the Non-Operative Treatment of Pediatric Supracondylar Humerus Fractures

**DOI:** 10.5812/atr.10570

**Published:** 2013-06-01

**Authors:** Seyed Ali Marashi Nejad, Seyed Abdolhossein Mehdi Nasab, Mohammad Baianfar

**Affiliations:** 1 Department of Orthopedics, Musculoskeletal and Rehabilitation Research Center, Ahvaz Jundishapur University of Medical Sciences, Ahvaz, IR Iran; 2 Department of Orthopedics, Nikan Hospital, Tehran, IR Iran

**Keywords:** Fractures, Supracondylar Humerus Fracture, Supination, Pronation

## Abstract

**Background:**

Supracondylar fracture of the humerus is the most common elbow injury that requires reduction and immobilization in the proper position to union. There are a few reports regarding the position of the forearm immobilization on elbow cosmetic outcome.

**Objectives:**

This study aimed to compare two modes of the forearm, supination and pronation in elbow deformity incidence after closed reduction and casting of this fracture.

**Patients and Methods:**

This prospective and descriptive study was carried out on children with supracondylar fracture of the humerus treated with closed reduction and cast immobilization in one of the two modes of either supination or pronation for a period of three weeks. Twenty-nine patients were immobilized in supination and 35 in pronation. Follow-up lasted for 8 months. Re-displacement was defined as the criteria and subsequent deformities of the elbow in patients, were assessed by clinical and radiographic examination.

**Results:**

A total of 64 patients, 50 boys and 14 girls, with the mean age of 4.8 years (3.1 to 8.5 years) participated. All fractures were closed and of the extension type. Forty-five cases had Gartland type II and 19 had type III fracture. Deformity of the elbow had occurred in seven cases (10.94%). Four cases of cubitus varus (CA 5 º - 16º) were observed in the supination group, of these, three patients had type III and one other had a type II fracture. In the pronation group, two cases of cubitus varus (CA 6 º - 14º) and one case of cubitus valgus (CA 17º) were observed, with type III initial fracture in all 3 cases.

**Conclusions:**

In regard to elbow malunion deformity, no obvious difference was observed between the two methods of supination and pronation in the closed treatment of supracondylar humerus fracture. However, as cubitus varus and valgus had occurred in both groups with unstable type III fractures, to prevent this complication, operative fixation is advised rather than closed reduction and position of the forearm immobilization.

## 1. Background

Supracondylar fracture of the humerus is the most common pediatric elbow injury in the first decade of life that occurs after falling with an outstretched arm. These fractures have two types, extension and flexion and 97 to 98% of cases are of the extension type, in which the distal humerus has a posterior displacement with a medial or lateral tilt. According to Gartland classification, this fracture is divided into 3 groups: 1) little or no displacement, 2) partial displacement with posterior periosteum intact and 3) total displacement (1-4). There are various modalities for the treatment of these fractures including closed reduction and casting, percutaneous pinning and open reduction and internal fixation. Closed reduction and casting in displaced supracondylar humerus fractures has been used with good results if performed within early hours of injury, preferably under general anesthesia and fluoroscop C-Arm. Re-reduction of fracture can be done within the first two weeks of fracture (5). The initial action in cases with displaced supracondylar fractures is closed reduction and immobilization using a splint or cast, with or without internal fixation. There has been concern about the forearm position of immobilization after closed reduction and also which mode, supination or pronation is more suitable to keep reduction and prevent further displacement. The choice is decided based on the medial or lateral displacement of distal humerus fracture and intact periosteum on each side. Abraham et al. described periosteum changes in the stability of the fracture and demonstrated that if the angle of fracture site is less than 40° and it is possible to flex the elbow more than 90° without radial artery pulse compromisation, then the fracture will be stable (6). Iqtedar et al. reported that for type III fractures, closed reduction and immobilization of the elbow in extension and supination was a safe method to preserve vascular status of the limb as well as maintaining the position of the reduction (5). In spite of the choice between the two modes, supination appears to be more comfortable for children.

## 2. Objectives

This study was conducted considering the prevalence of elbow deformity in patients who can be managed by closed treatment based on the position of supination or pronation for immobilization of the extremity.

## 3. Patients and Methods

This prospective descriptive and cross-sectional study was conducted from 2006 to 2009 in Imam Khomaini and Razi hospitals in Ahvaz, Iran. Inclusion criteria were supracondylar humerus fracture in pediatrics that could be treated with closed reduction and cast immobilization. Exclusion criteria were the following: open fracture, fractures that required surgery for any other reason such as, vascular damage, multiple fractures, floating elbow, inability to gain acceptable reduction and re-displacement during cast immobilization or failing to attend follow-ups. Closed reduction was performed in the plaster emergency room or operating theater under anesthesia. The upper arm was held by an assistant in the supine position and longitudinal traction was applied on the forearm, then manipulation and reduction of postero medial or postero lateral displacement of the distal fragment was done. To control reduction, C-arm fluoroscopy or portable radiography was used. The elbow was flexed up to the point where radial pulse was still palpable. After close reduction, plaster cast was applied to the forearm in supination in the first and pronation in the second group. Radiographic monitoring was performed every week for three weeks. If an unacceptable displacement that needs surgery was observed within seven to ten days, the patient was excluded from the study. Union was confirmed by observing callus tissue, fading fracture line, and lack of pain. After three weeks, plaster splint was removed, active motion was allowed and physiotherapy was advised. Clinical and radiographic examinations for the elbow’s appearance, humeral-ulnar or carrying angle (CA) were performed. This study was approved by the Ethics committee of our university and an informed consent was taken from the parents of the children. All the patients were followed up for 8 months. Analysis of statistical data was performed initially by preparing a cod-sheet from the data collected, and entering in the SPSS-software version 13. For comparisons of qualitative statistics Exact Fisher and Chi-square tests were performed and for quantitative statistics, t-test was used. P ≤ 0.05 were regarded significant.

## 4. Results

Sixty-four patients participated in the study (50 boys and 14 girls) with the mean age of 4.8 years in both groups (3.1 - 8.5 years). A total of 38 patients had fractures on the left and 26 on the right elbow. There was no significant difference between the two groups with regard to the average age at the time of fracture and side of involvement (P > 0.05), but with respect to gender, difference was significant (P = 0.035). Twenty-nine fractures were immobilized in supination and the 35 other in pronation. The number of patients and other details according to Gartland classification are presented in Table 1. In the supination group, 4 cases of cubitus varus (CA 5º - 16º, mean = 12º) were observed. One patient had anterior angulation about 14 deg and loss of 25 deg in elbow flexion. In the pronation group, there were 2 cases of cubitus varus (CA 6º - 14º deg, mean = 11º) and 1 case of cubitus valgus (CA 17°) compared with a contralateral healthy elbow. Out of the 7 cases with an elbow with malunion deformity, 6 cases (85.8%) had type III and 1 case (14.2%) had type II fractures with a significant difference P = 0.006 (Table 2).

**Table 1.** Patients’ Details According to the Position of Forearm Supination and Pronation of Casting

Variable	Pronation No. (n=29)	Supination No. (n=35)
**Gender**		
Male	23	27
Female	6	8
**Affected side**		
Right	11	15
Left	18	20
**Mean age, y**	4.3	4.6

**Table 2.** Results in Both Groups of Patients

Variable	Pronation, No. (n =29)	Supination, No. (n = 35)
**Type of fracture (Gartland classification)**		
II	21	24
III	8	11
**Cubitus varus**		
Type II	0	1
Type III	2	3
**Cubitus valgus**		
Type II	0	0
Type III	1	0

There was no significant difference between the two groups with regard to the incidence of elbow deformity as a malunion complication P > 0.05. Regarding the type of deformity, 6 cases of cubitus varus and one case of cubitus valgus were observed that showed significant differences P = 0.035. Volkmann's ischemic contracture did not occur in any type II or III fractures. One patient had an ulnar nerve and 1 other had a median nerve injury after cast removal. Both of them had type III fracture and had recovered after four months.

## 5. Discussion

Studies on the biomechanics of supracondylar humerus fractures indicate that the extension type with posterior or posteromedial displacement of the distal fragment is the most common type. In this case, the medial periosteum remains intact and under tension (4, 7, 8). It acts like a hinge, and with the forearm in pronation, this hinge stiffens, which helps to preserve the reduction. This is an important point by which the fracture is treated with closed reduction. The most common deformity of this fracture is cubitus varus with a risk of 9% to 58% in closed fractures (4). Reynolds reported this deformity in up to 14% (9), and Pirone et al. reported this incidence in 7.9% of 101 patients who were treated with plaster splint, and 1.9% in 105 patients with percutaneous pin fixation (10). Shoaib reported better results with plaster splint in type II fractures than in type III (11) which agree with the results of our study. Since in recent years, surgery has been used more frequently for displaced supracondylar fractures, deformity of the elbow is less observed (12). A cubitus varus malunion, usually affects the appearance of the elbow, but not its function, except where joint instability or damage to ulnar nerve occurs (13). Given that there is a normal valgus angle in the elbow which is the carrying angle, cubitus valgus deformity has a more acceptable appearance, but may lead to delayed ulnar nerve palsy (14). In this study, a total of six cases of cubitus varus and one case of cubitus valgus with carrying angle of 17° were observed, and these appeared to be due to impaired epiphysis growth, mal-union, or gradual displacement during cast immobilization when the elbow swelling subside. However, the exact cause was not identified. In the past, it was thought that elbow deformity may be caused by osteonecrosis of the medial trochlea or impaired epiphysis growth of distal humerus. However, this belief is no longer accepted, and mal-union is thought to be the real cause, and asymmetric development is blamed to a less degree. We think that the deformity occurred gradually and we could observe it when the patients had a complete bony union and were able to have more extension. Therefore, attention to the quality of the reduction and appropriate fixation or open reduction with pinning is of crucial importance in avoiding varus and valgus deviations, particularly in unstable type III fractures. Given that elbow deformity in both supination and pronation after closed reduction of supracondylar humerus fracture was the same, it seems that the type of position does not cause deformity. In fact, it is the fracture type and reduction quality that are important. Deformity of the elbow occurred in type III fractures, which are unstable, and out of seven patients, six cases (85.8%) had type III fractures. Varus and valgus deformity in the elbow does not correct spontaneously and in cases with severe cosmetic deformity, malfunction of the elbow or tardy ulnar nerve palsy, a corrective supracondylar osteotomy will be required. So it is important to handle type III fractures with special care and strict follow-ups, when non operative treatment has been used. Percutaneous pin or open reduction and internal fixation may be considered as a more appropriate method for this fracture to prevent elbow deformity (14). One of the limitations of our study was applying this treatment for type III unstable fractures, in which re-displacement may occur during the first few weeks of treatment. Despite an acceptable initial reduction, this may lead to malunion of the fracture in the final stages of union. Another limitation was that the study was performed in a short term follow up. Because deformity may have some remodeling potentials and even worsen with time, a retrospective long-term follow-up is advised. In conclusion, After non operative treatment of the pediatric supracondylar fractures, cubitus varus was a more frequent complication than cubitus valgus. Cubitus varus was observed more frequently in patients who were treated with forearm in supination, while cubitus valgus was seen after pronation, although this difference was not significant. Both of these complications were seen after type II and III fractures which are unstable.
